# A prospective, double-blind, randomized controlled trial of the angiotensin-converting enzyme inhibitor Ramipril In Aortic Stenosis (RIAS trial)

**DOI:** 10.1093/ehjci/jev043

**Published:** 2015-03-21

**Authors:** Sacha Bull, Margaret Loudon, Jane M. Francis, Jubin Joseph, Stephen Gerry, Theodoros D. Karamitsos, Bernard D. Prendergast, Adrian P. Banning, Stefan Neubauer, Saul G. Myerson

**Affiliations:** 1 University of Oxford Centre for Clinical Magnetic Resonance Research (OCMR), Division of Cardiovascular Medicine, Radcliffe Department of Medicine, John Radcliffe Hospital, Headley Way, Headington, Oxford OX3 9DU, UK; 2 Centre for Statistics in Medicine, University of Oxford, Nuffield Orthopaedic Centre, Oxford OX3 7LD, UK; 3 Department of Cardiology, Oxford Heart Centre, John Radcliffe Hospital, Oxford OX3 9DU, UK

**Keywords:** aortic stenosis, ACE inhibition, myocardium, valve disease

## Abstract

**Aims:**

Angiotensin-converting enzyme (ACE) inhibitors improve left ventricular (LV) remodelling and outcome in heart failure and hypertensive heart disease. They may be similarly beneficial in patients with aortic stenosis (AS), but historical safety concerns have limited their use, and no prospective clinical trials exist.

**Methods and results:**

We conducted a prospective, randomized, double-blind, placebo-controlled trial in 100 patients with moderate or severe asymptomatic AS to examine the physiological effects of ramipril, particularly LV mass (LVM) regression. Subjects were randomized to ramipril 10 mg daily (*n* = 50) or placebo (*n* = 50) for 1 year, and underwent cardiac magnetic resonance, echocardiography, and exercise testing at 0, 6, and 12 months, with follow-up data available in 77 patients. There was a modest but progressive reduction in LVM (the primary end point) in the ramipril group vs. the placebo group (mean change −3.9 vs. +4.5 g, respectively, *P* = 0.0057). There were also trends towards improvements in myocardial physiology: the ramipril group showed preserved tissue Doppler systolic velocity compared with placebo (+0.0 vs. −0.5 cm/s, *P* = 0.04), and a slower rate of progression of the AS (valve area 0.0 cm^2^ in the ramipril group vs. −0.2 cm^2^ in the placebo arm, *P* = 0.067). There were no significant differences in major adverse cardiac events.

**Conclusion:**

ACE inhibition leads to a modest, but progressive reduction in LVM in asymptomatic patients with moderate–severe AS compared with placebo, with trends towards improvements in myocardial physiology and slower progression of valvular stenosis. A larger clinical outcome trial to confirm these findings and explore their clinical relevance is required.

## Background

Aortic stenosis (AS) is the most common form of valvular heart disease in the Western world affecting 5% of those aged over 75 years.^1^ The standard treatment once symptoms or LV dysfunction develops is aortic valve replacement (AVR).^2^ However, the perioperative risks of this procedure rise with increasing age and co-morbidity.^3,4^ Medical therapy to delay the onset of symptoms and progression of AS would be highly desirable, but to date, no medical therapy has been shown to be beneficial in patients with AS. Early retrospective studies of statins suggested that the progression of AS could be delayed,^5,6^ but subsequent larger randomized trials were negative,^7–9^ underlining the importance of prospective trials.

The response of the myocardium is likely to be as important as the degree of valve stenosis,^10^ and both the aortic valve area^11^ and measures of the myocardial response [degree and pattern of left ventricular hypertrophy (LVH),^12^ presence of fibrosis,^13^ reductions in longitudinal strain^14^] have been shown to determine prognosis in these patients.

The renin–angiotensin system (RAS) has a major influence on myocardial physiology, and there is some evidence for this in AS: it regulates the degree of LVH,^15^ the extent of fibrosis in the myocardium,^16^ and may even play a role in aortic valve thickening.^17^ RAS inhibitors reduce LVM independent of blood pressure (BP) (suggesting a direct myocardial effect),^18^ can reduce the extent of myocardial fibrosis,^19^ and have been shown to improve clinical outcome through LV remodelling in other disease areas—e.g. post-myocardial infarction,^20^ heart failure,^21^ and hypertension-induced LVH.^18^ Inhibition of the RAS with angiotensin-converting enzyme inhibitors (ACEi) would therefore seem an attractive option to improve LV remodelling and myocardial physiology in AS, resulting in better tolerance to the valve obstruction, potentially delaying the onset of symptoms and reducing need for aortic valve surgery. ACEi have, however, been traditionally regarded as contraindicated in moderate or severe AS due to the theoretical danger of syncope caused by afterload reduction, and current guidelines still advise caution. There are however no clinical studies indicating harm, and in fact, the limited animal and human data that exist do not suggest harm, and even suggest benefit.^22–24^ Recent retrospective studies suggest a potential benefit in AS patients taking ACEi or ARBs^25^ as well as reductions in the progression of AS,^26^ but these studies are subject to significant selection and other biases, in a similar way to the early retrospective statin studies in AS, and a prospective clinical trial is required.

With the Ramipril In Aortic Stenosis (RIAS) trial, we therefore sought to carry out the first prospective, randomized, placebo-controlled study of ramipril in AS. Given the perceived historical problems of ACEi in this population and the large scale required for a clinical outcome trial, we planned an intermediate (physiological) study to determine whether there were any positive physiological changes, and an absence of harm, before embarking on a large-scale clinical outcome study. The aims of this study were as follows:

1. To examine changes in myocardial physiology, in particular the regression of left ventricular mass (LVM), as well as other LV physiological parameters (perfusion, LV strain, fibrosis) using multi-parametric cardiac magnetic resonance (CMR) in patients with moderate to severe AS.
2. To assess the safety and tolerability of ramipril in these patients.
3. To examine potential improvements in effort tolerance.

## Methods

The study protocol and detailed methods have been previously published,^27^ and a concise summary is provided here. The protocol was approved by the Oxfordshire research ethics committee C (reference 07/H606/139) and the Medicines and Healthcare products Regulatory Agency (clinical trial authorization 21584/0226/001-004). The trial was also registered with the European Community clinical trials database (EudraCT no 2007-005224-32) and the ISRCTN register (24 616 095). All patients gave their written informed consent.

### Study population

Subjects were recruited from clinics at the John Radcliffe Hospital and surrounding institutions. All patients aged >18 years with moderate or severe AS by standard echocardiographic criteria [valve area <1.5 cm^2^, or peak velocity >3.0 m/s (peak valve gradient >36 mmHg)],^2^ who were asymptomatic as judged by patient-reported symptoms, and who did not have indications for valve replacement surgery were invited to participate. All had normal LV function (ejection fraction >50% by echocardiography) and no other significant (>mild) valvular heart disease, excess hypo- or hypertension (BP < 100/40 or >200/110 mmHg). Intolerance of ACEi or angiotensin receptor blockers (ARBs) or their prescription over the previous 3 months were also exclusion criteria.

### Study design and drug titration schedule

**Figure 1:**
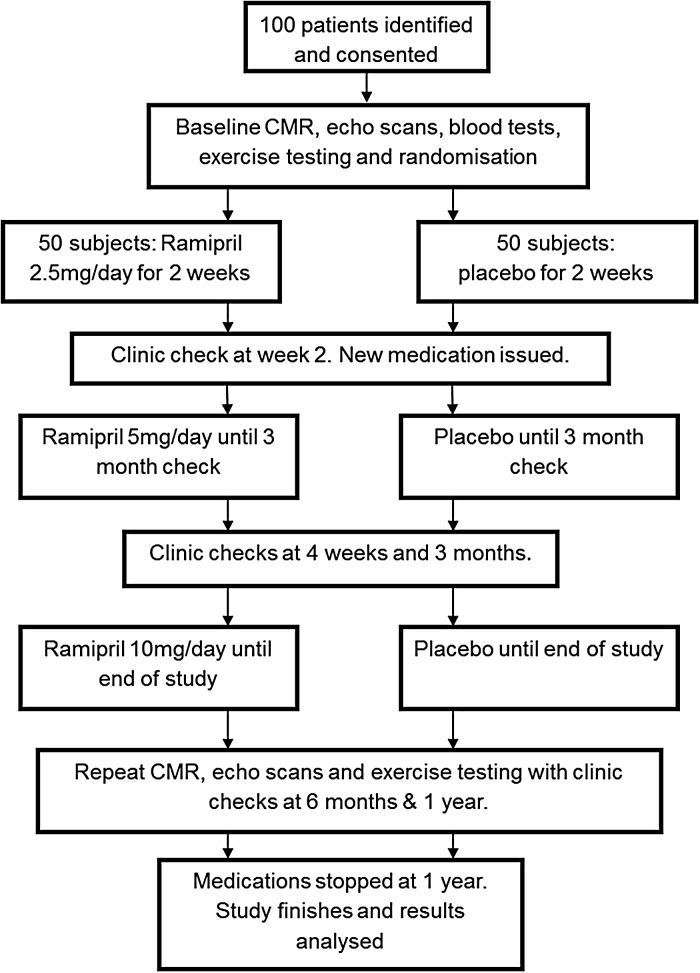
Study design and flow chart.

This was a randomized, double-blind, placebo-controlled trial to assess physiological changes in the myocardium with ramipril; it was not powered for clinical end points. *Figure 1* summarizes the study design. After baseline visits, subjects were randomized to ramipril or placebo for 1 year. An initial pack of study medication (ramipril 2.5 mg daily or placebo) was provided for 2 weeks to ensure no adverse symptoms. The ramipril was then increased to 5 mg daily and again to 10 mg daily at 12 weeks. The occurrence of adverse events and any changes in laboratory parameters were noted throughout the study. Full assessments occurred at baseline, 6 months and 1 year, and researchers were blinded to the randomization until after data analysis by the statisticians. The primary outcome was the change in LVM from baseline to 12 months measured by CMR. Secondary end points included changes in left ventricular ejection fraction (LVEF), change in other myocardial functional parameters assessed by CMR (perfusion, T1 values, strain), and echocardiography (including diastolic parameters); change in B-type natriuretic peptide (BNP); and change in distance walked on ETT.

### Cardiovascular magnetic resonance

Patients were scanned using a 1.5-T Avanto CMR system (Siemens, Erlangen, Germany). Comprehensive CMR assessment was carried out at baseline and 1 year, while at 6 months only LV volumetric analysis was performed to determine the time course of any changes in the primary end point. LVM, LV volumes, and LV function were assessed using a stack of steady-state free precession short-axis cine images, in accordance with the Society for Cardiovascular Magnetic Resonance guidelines.^28^ The aortic valve was imaged using short-axis steady-state free precession (SSFP) cine sequences at the valve tips in mid-systole, and transvalvular velocity was measured using breath-hold through-plane phase-contrast velocity mapping just distal to the aortic valve (at the vena contracta).

Myocardial strain was assessed using a grid-based ‘tagging’ sequence^29^ in the horizontal long-axis (four chamber) and three short-axis views (basal, mid-ventricular, apical), each during a single breath hold. Diffuse myocardial interstitial fibrosis was assessed using non-contrast myocardial T1 mapping, as a surrogate marker for this in AS.^30^ A Short Modified Look Locker Inversion recovery (ShMOLLI) technique was used,^31^ in a single mid-ventricular slice, with the assumption that the degree of diffuse fibrosis was similar throughout the myocardium, and the average T1 value over this whole slice was calculated as previously described.^30^ Late gadolinium enhancement (LGE) imaging was also performed to assess more patchy, confluent areas of fibrosis 10 min after the injection of gadolinium for perfusion (see below) using a standard inversion recovery technique.^32^ This was repeated with the phase-encoding direction swapped to exclude artefact.

Myocardial perfusion reserve was assessed according to guidelines,^33^ following administration of adenosine at a rate of 140 µg/kg/min for 3 min. Gadolinium-based contrast (Omniscan 0.03 mmol/kg at 6 mL/s; Nycomed Amersham, Little Chalfont, UK) was administered intravenously, to maintain a linear relationship between signal intensity and perfusion. Perfusion imaging was performed in three short-axis sections during the first pass of the contrast bolus^34^ and repeated at rest at least 20 min later. Myocardial perfusion reserve index was calculated for all 16 segments, and the average value was used (MPRI: the ratio of stress to rest normalized myocardial perfusion upslopes).

### Echocardiography

Transthoracic echocardiography was carried out, particularly to assess diastolic function, using a Philips iE33 advanced echo system (Philips Medical Systems, Best, Netherlands). A full echo study was performed according to guidelines for assessment and classification of AS and chamber quantification, including Tissue Doppler measurements of systolic tissue deformation.^2,35^ LV diastolic function was assessed using tissue Doppler measurements of medial and lateral mitral annular velocities in early diastole and mitral valve inflow.^35,36^

### Exercise treadmill testing

Exercise testing was carried out to assess the maximum walking distance as a continuous variable in the study population (6 min walk tests are less useful in active patients). The Naughton protocol was chosen^37^ as it contains multiple small increments in speed and incline, to provide a smooth increase in workload more applicable to a continuous variable than fewer large increases in workload, which occur in other protocols. The test was carried out under medical supervision with continuous 12-lead electrocardiogram recording and regular BP monitoring.

### Clinical events

These were not a primary aim of this study, which was too small to examine this robustly, but any major adverse cardiac events (death, AVR or hospital admission with cardiac symptoms) were recorded. Any decision to refer patients for AVR was taken by the patient's treating cardiologist, who was also blinded to the study medication.

### Statistical methods

A full description of these is published with the trial protocol,^27^ and a brief summary is included here. Analyses were carried out by an independent statistician (SG) at the Centre for Statistics in Medicine, University of Oxford in accordance with the trial statistical analysis plan. The sample size was based on changes in LVM, using a baseline from a prior study of AS with CMR (142 ± 35 g/m^2^),^38^ and a 15% (21.3 g/m^2^) reduction in LVM—the mean change from a large meta-analysis of antihypertensive treatments.^18^ We used a one-sided test (only including reduction in LVM) with 85% power (β error) and 95% confidence (α error). The number needed in each study group with these calculations was 43, and a total of 50 patients per group was planned, allowing for a 15% drop-out rate. Primary and secondary analyses were conducted on the modified intention to treat (mITT) population, including all participants who received study medication and at least one follow-up measurement.

## Results

**Figure 2:**
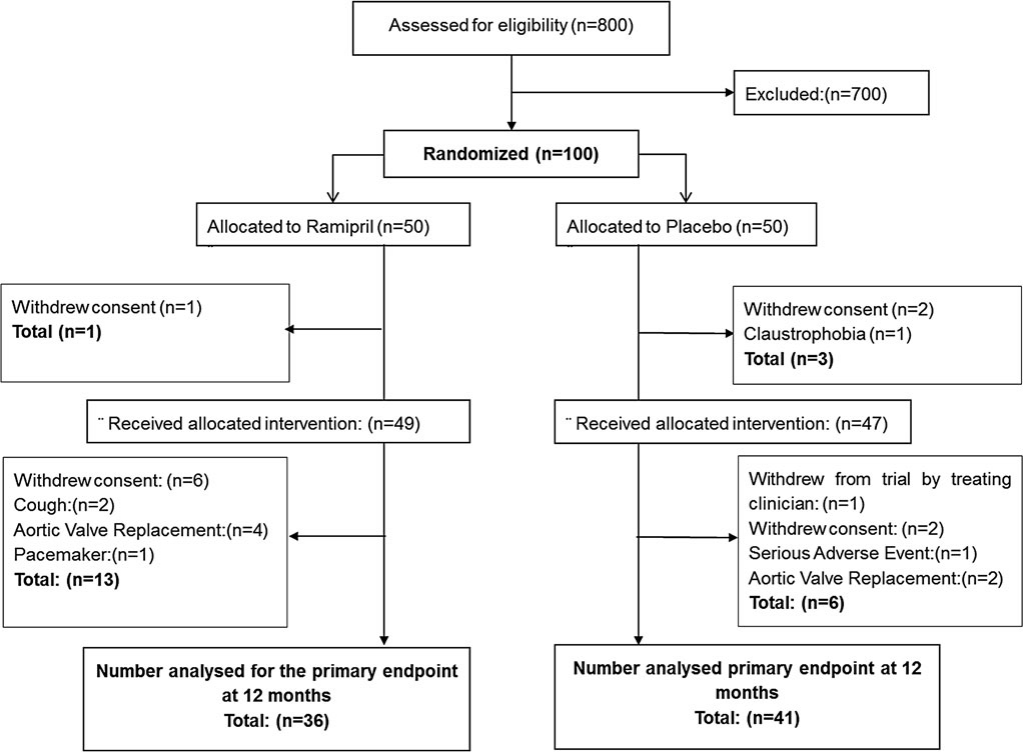
CONSORT flow chart—numbers of subjects randomized and allocated to each treatment.

One hundred patients with moderate (*n* = 80) or severe (*n* = 20) AS were recruited between October 2008 and April 2011, and baseline patient characteristics are summarized in *Table 1*. Four patients withdrew after randomization, leaving 96 patients who received the trial medication (47 placebo; 49 ramipril). Nineteen patients withdrew during the trial (mostly within the first 6 months), leaving 77 patients who completed the 1-year assessment—only 2 patients (one from each group) withdrew between 6 and 12 months; see *Figure 2* for CONSORT diagram. Treatment groups were balanced at baseline with respect to demographics, symptoms, CMR, echocardiography, and exercise testing data (*Table 1*). Any differences were slight and the numbers too small to draw any inferences.

**Table 1:** Baseline patient characteristics

Characteristics	Placebo group (*n* = 47)	Ramipril group (*n* = 49)	*P*-value (unpaired *t*-test)
Male gender, *n* (%)	36 (75.0)	35 (71.4)	0.70
Age	70.0 (14.6)	67.2 (13.7)	0.34
BMI	28.0 (5.4)	29.2 (4.8)	0.27
Systolic BP (mmHg)	135 (18)	130 (16)	0.13
Diastolic BP (mmHg)	77 (9)	77 (6)	0.57
Smoker, *n* (%)	3 (6.1)	5 (10.0)	0.48
Ex-smoker, *n* (%)	8 (16.7)	2 (4.1)	0.04
Hypertension, *n* (%)	17 (35.4)	11 (22.4)	0.16
Myocardial infarction, *n* (%)	1 (2.1)	0 (0.0)	0.32
CABG, *n* (%)	2 (4.2)	2 (4.3)	0.98
Stents, *n* (%)	2 (4.2)	2 (4.1)	0.98
Diabetes, *n* (%)	2 (4.2)	1 (2.0)	0.55
Medications			
β-Blockers, *n* (%)	10 (20.8)	10 (20.8)	0.80
Statins, *n* (%)	27 (56.3)	20 (41.7)	0.16
Aspirin, *n* (%)	16 (33.3)	20 (41.7)	0.40
Diuretics, *n* (%)	10 (20.8)	5 (10.4)	0.04
Calcium channel blockers, *n* (%)	10 (20.8)	5 (10.4)	0.16
CMR parameters			
LVM (g)	154.8 (40.0)	158.4 (49.8)	0.80
LVM index (g/m^2^)	80.7 (19.5)	79.6 (20.5)	0.80
LVEF (%)	72.6 (8.2)	70.8 (8.0)	0.27
Peak velocity (m/s)	3.2 (0.6)	3.1 (0.7)	0.33
Aortic valve area (cm^2^)	1.2 (0.4)	1.2 (0.3)	0.59
Number with moderate AS (%)	37 (79%)	39 (80%)	0.85
T1 value (ms)	961 (39)	951 (24)	0.35
MPRI	1.20 (0.41)	1.37 (0.37)	0.13
Longitudinal strain (%)	−9.7 (2.4)	−9.7 (2.9)	0.17
Circumferential strain (%)	−15.9 (4.5)	−17.1 (2.2)	0.17
Echo parameters			
AV max (m/s)	3.5 (0.5)	3.3 (0.5)	0.12
AV mean (m/s)	2.4 (0.4)	2.3 (0.4)	0.09
Septal *E*/*E*' ratio	13.0 (5.7)	12.5 (4.8)	0.72
Lateral *E*/*E*' ratio	11.0 (5.3)	10.1 (5.7)	0.52
S-wave (cm/s)	6.3 (1.3)	6.2 (0.8)	0.85
Biomarkers			
BNP (pmol/L)	20.9 (35.5)	14.9 (21.5)	0.33
Exercise tolerance			
Exercise distance (m)	985 (360)	1030 (386)	0.57

Values are mean (standard deviation) unless indicated otherwise.

BMI, body mass index; LVM, left ventricular mass; LVEF, left ventricular ejection fraction; MPRI, myocardial perfusion reserve index; BNP, B-type natriuretic peptide.

### Primary end point: change in LVM

**Figure 3:**
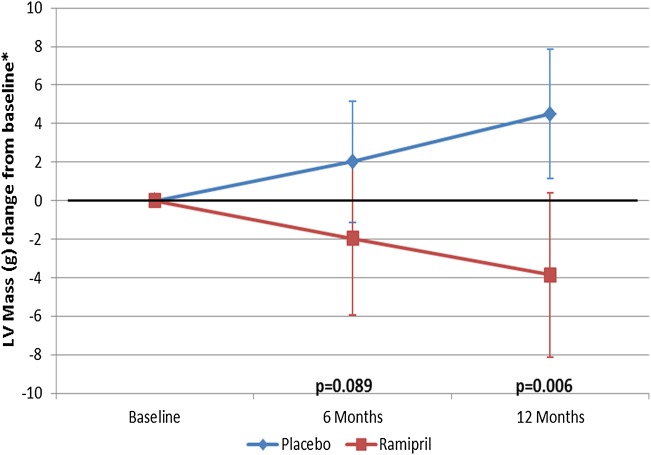
Changes in LV mass with ramipril 10 mg daily or placebo over a 12-month period.

There was a modest but significant difference between treatment groups at 1 year: the ramipril group showed a reduction in mean LVM of −3.9 g compared with an increase of +4.5 g in the placebo group, leading to an overall mean difference of 8.4 g (*P* = 0.0057, *Figure 3*). The change in LVM was also progressive, with a similar reduction in LVM with ramipril and increase with placebo at 6 months, though with half the degree of change that occurred at 1 year: mean difference between groups 4.0 g (*P* = 0.089).

### Secondary end points

#### Other parameters of LV physiology

Most assessments of myocardial physiology using CMR and echocardiography did not show significant differences between treatment groups (*Table 2*). This included LVEF, myocardial T1 values (as a surrogate marker of interstitial fibrosis), perfusion indices, strain, and diastolic function. Systolic myocardial velocity (measured using tissue Doppler S-wave) worsened slightly in the placebo group compared with ramipril (−0.5 vs. 0.0 cm/s, *P* = 0.04). However, these group differences are small and baseline S-wave measurements were lower than normally expected, so this may reflect measurement variability.

**Table 2:** Primary and secondary end points

	Placebo^a^	Ramipril^a^	Difference^b^	*P*-value
	*n* = 41	*n* = 36		
Primary end point				
Change in LVM (g)				
6 months	+2.0 ± 1.6	−2.0 ± 1.7	−4.0 ± 2.3	0.089
12 months	+4.5 ± 2.1	−3.9 ± 2.2	−8.4 ± 3.0	**0**.**006**
Change in LVM index (g/m^2^)				
6 months	+1.3 ± 0.9	−1.0 ± 1.0	−2.3 ± 1.3	0.078
12 months	+3.5 ± 1.5	−1.0 ± 1.6	−4.4 ± 2.1	**0**.**036**
Secondary end points				
Change in CMR parameters				
LVEF (%)	−0.3 ± 1.0	+1.0 ± 1.0	1.3 ± 1.3	0.328
AV_max_ (m/s)	+0.1 ± 0.1	0.0 ± 0.1	−0.1 ± 0.1	0.277
Aortic valve area (cm^2^)	−0.2 ± 0.05	0.0 ± 0.1	−0.2 ± 0.1	0.067
T1 values (ms)	−2 ± 6	+4 ± 7	6 ± 9	0.530
MPRI^c^	+0.1 ± 0.1	0.0 ± 0.1	−0.1 ± 0.2	0.518
Longitudinal strain (%)	+1.0 ± 0.7	+0.4 ± 0.7	−0.6 ± 1.0	0.550
Circumferential strain (%)	+0.1 ± 0.8	+0.7 ± 0.8	+0.6 ± 1.1	0.584
Change in echo parameters				
AV_max_ (m/s)	+0.03 ± 0.49	+0.05 ± 0.30	−0.025 ± 0.10	0.801
AV mean (m/s)	+0.04 ± 0.30	+0.05 ± 0.25	−0.012 ± 0.06	0.841
Septal *E*/*E'*	+0.7 ± 1.1	+1.6 ± 1.1	+0.9 ± 1.6	0.530
Lateral *E*/*E'*	+0.4 ± 1.1	+1.2 ± 1.4	+0.8 ± 1.8	0.632
S-wave (cm/s)	−0.5 ± 0.2	0.0 ± 0.2	+0.5 ± 0.2	**0**.**040**
Change in other parameters				
Systolic BP (mmHg)	−2.9 ± 2.1	−5.5 ± 2.2	−2.7 ± 3.0	0.374
Diastolic BP (mmHg)	−1.4 ± 1.1	−3.6 ± 1.8	−2.2 ± 1.6	0.160
BNP (pmol/L)	+8.2 ± 3.4	−0.5 ± 3.7	−8.6 ± 5.1	0.086
Exercise distance (m)	+29 ± 25	−20 ± 26	−49 ± 36	0.176

*P*-values in bold indicate *P* < 0.05. Abbreviations as in *Table 1*.

^a^Mean change from baseline, adjusted for baseline.

^b^Result of linear regression assessing change from baseline, adjusted for baseline.

^c^*n* = 30 for this parameter.

#### Aortic valve area (by CMR direct planimetry)

At 1 year, there was a trend towards a slower rate of progression of AS in the ramipril group, with a static aortic valve area (0.0 cm^2^) compared with a reduction in the placebo group (−0.2 cm^2^), *P* = 0.067. This did not, however, impact on the peak velocity across the aortic valve which did not differ significantly between the ramipril and placebo groups: change at 1 year +0.03 and +0.12 m/s, respectively, *P* = 0.28.

#### Changes in BNP

There was a trend towards stabilization in BNP in the ramipril group (−0.50 pmol/L) and increase in the placebo group (+8.2 pmol/L), but the difference between groups was not statistically significant (*P* = 0.086), and these changes were very small, which, coupled with the low mean baseline values (15–21 pmol/L), limits any interpretation.

#### Changes in exercise tolerance

There was no significant difference in the mean change in distance walked on the treadmill at 12 months compared with baseline (−20.1 m in the ramipril group vs. +28.7 m in the placebo group; *P* = 0.18).

#### Blood pressure

BP reduced slightly in both groups at 12 months, with systolic pressure falling by −5.5 vs. −2.9 mmHg for ramipril and placebo, respectively, though differences between groups were not statistically significant (*P* = 0.37).

### Adverse events

Ramipril was well tolerated. There was one serious adverse event in a patient who developed neutropenia, leading to discontinuation of the trial medication (which was placebo). The trial was not powered for clinical events, and there were the same number of major adverse cardiac events (5) in each group, and a similar number of AVRs: ramipril 4 vs. placebo 2; *P* = 0.52.

## Discussion

In the first prospective, randomized, placebo-controlled trial of ACEi in patients with moderate and severe asymptomatic AS, we have shown that ramipril reduces the hypertrophic response of the myocardium, may have additional benefits, and is well tolerated.

### LV response to ramipril in AS

We found a modest reduction in LVM with ramipril, and although the observed differences are small, these represent group mean differences, suggesting an overall shift in LVM within the cohort. There was also a progressive change over the year (*Figure 3*), which may have continued if ramipril had been given for longer, and these changes occurred despite the fixed outflow tract obstruction from the AS. There were small differences in BP between the groups at 12 months, but the differences were not statistically significant, suggesting that this reduction in mass was driven by direct effects of ramipril on the myocardium, similar to the benefits described in the hypertensive population,^39^ though a contribution from the BP is difficult to rule out given the moderate group sizes. Baseline LVM index was also significantly smaller than reference group used for power calculations (80 vs. 142 g/m^2^, respectively), but the previous study^38^ examined patients just prior to AVR, and it is likely that these were at the more severe end of the spectrum, which may explain the difference (our study included many patients with moderate AS). Patients with severe LVH from non-valvular causes have an adverse prognosis,^12^ and reductions in LVM have been associated with improvements in prognosis.^40^ Reduced LVH in response to pressure overload in AS might therefore lead to improvements in prognosis, and some studies suggest a stronger prognostic association with LVM than the severity of AS *per se.*^12^ It would thus be reasonable to hypothesize that ramipril may lead to improved prognosis in AS by reducing LVH, and this is supported by findings from a retrospective study that showed reduced mortality in patients taking ACEi in AS.^25^ The current study was not powered for clinical outcomes however, and any prognostic benefit from RAS inhibition in AS requires a larger clinical outcome trial to address this question.

Other CMR measures of LV physiology—perfusion, strain, T1 values (an indirect CMR measure of interstitial cardiac fibrosis^30^)—or presence of LGE did not differ between groups for the most part. There were small improvements in the echocardiographic tissue Doppler S-wave (a measure of longitudinal contraction) with ramipril, though baseline values were lower than normal—this may reflect reduced long-axis contraction in established AS and would be in keeping with the reduced longitudinal strain values in both groups, but it could also be partly due to mitral annular calcification restricting systolic mitral annular velocities (this is relatively common in AS and was not assessed in our study). There were also no differences in exercise capacity between groups, either at baseline or any changes during the trial, in keeping with small, if any, differences in myocardial physiological parameters. All patients had normal ejection fractions at baseline, however, which may partly explain the lack of a consistent beneficial effect on myocardial physiology with ramipril (it may have been difficult to demonstrate improvements in normal function), though the study may also be under-powered to show changes in these parameters.

We hypothesized at the outset that ramipril might improve interstitial cardiac fibrosis by lowering circulating angiotensin II, a promoter of interstitial cardiac fibrosis,^41^ and losartan has previously been shown to cause regression of interstitial cardiac fibrosis and improvements in diastolic function in hypertensive patients.^19^ Our inability to demonstrate changes in T1 values (a surrogate marker of interstitial fibrosis^30^) may have been due to the imaging technique being insufficiently sensitive to pick up small changes in interstitial fibrosis. Alternatively, the reduction in LVM may be due to a reduction in myocyte size, as recently shown in patients post-AVR,^42^ and there may have been little, if any, regression of fibrosis. Further, in other studies, the regression in cardiac fibrosis in hypertensive patients (associated with improvements in diastolic parameters) was only seen in those with *severe* interstitial fibrosis—no changes in either fibrosis or diastolic function were seen in those with mild or moderate fibrosis.^19^ As the majority of patients in our cohort had moderate AS (in whom we have previously shown T1 values did not differ from normal controls^30^), the fibrosis burden may have been too light for significant changes with medical treatment to be demonstrated.

Our study did not show any significant improvement in perfusion indices, despite myocardial perfusion previously being associated with LVM in patients with severe AS.^43^ The number of patients who completed perfusion imaging at both time points in our study was small however (*n* = 30), and the reduction in LVM may have been too small and the relationship between LVM and perfusion too weak to show any changes. This relationship between perfusion and increased LVM may also differ in moderate AS (which represented the majority of our cohort).

### Potential effect on the aortic valve

The ramipril group showed a trend towards reduced progression of the AS (change in valve area 0 vs. −0.2 cm^2^ in the ramipril and placebo groups respectively, though the statistical strength of the difference was weak (*P* = 0.067). There is nonetheless some evidence to support the involvement of the RAS in aortic valve calcification. Activation of the local RAS in aortic valves has been seen in AS;^44,45^ angiotensin II is implicated in aortic valve thickening;^17^ and aortic valve weights (extracted at the time of AVR) are significantly lower in AS patients taking ARBs.^46^ Retrospective clinical studies show conflicting results however: O'Brien *et al.*^47^ reported significant reductions in aortic valve calcification in AS treated with ACEi, while Rosenhek *et al.*^5^ found no change in the progression of aortic valve disease or calcification. The ongoing ROCK-AS trial (*NCT00699452*) aims to analyse the degree of valvular inflammation, calcification, lipid accumulation, and fibrosis from histology of aortic valves removed at surgery from AS patients taking candesartan compared with placebo and may help to answer these questions.

### Clinical events and safety

There were no differences in the progression to AVR or major adverse clinical events between the two groups. However, the trial was not powered for clinical events, consequently the number of events was small and the length of time too short for any meaningful conclusions. Encouragingly, there was no increase in adverse events for patients taking ramipril, which supports the hypothesis that ACEi are safe in AS, as suggested by a number of small retrospective studies.^23,24^ Our group size was modest though, and a larger study with longer follow-up would be required to evaluate these aspects more robustly.

### Limitations

The data in this study are encouraging but were based on a relatively small sample size, with limited follow-up of 1 year. It assessed detailed physiological changes, which are feasible in this sample size with CMR, but the study was significantly under-powered for any assessment of clinical outcome. A further clinical outcome study would be required to assess this, and preliminary power calculations suggest that such a study would require around 1400 subjects over 4 years to determine any significant effect on clinical events.

## Conclusion

In this first prospective trial of ACEi in patients with severe and moderate AS, we demonstrated that they are likely to be well tolerated and may reduce LVH. The study size was modest however, and a larger trial powered for clinical outcomes is required to confirm these physiological changes and determine whether they translate into improved clinical outcomes. If this is shown, ACEi could potentially be of benefit to significant numbers of patients with asymptomatic AS and would be the first medical treatment for this condition.

## Funding

The work was supported by a Heart Research UK project grant RG2512 and the Oxford Comprehensive Biomedical Research Centre, funded by the UK National Institute for Health Research (A.P.B., B.D.P., S.N., S.G.M.). S.B. was supported by a British Heart Foundation Clinical Research Training Fellowship FS/10/015/28104. Funding to pay the Open Access publication charges for this article was provided by the University of Oxford.
